# Estimating the impact of control measures to prevent outbreaks of COVID-19 associated with air travel into a COVID-19-free country

**DOI:** 10.1038/s41598-021-89807-y

**Published:** 2021-05-24

**Authors:** Nick Wilson, Michael G. Baker, Tony Blakely, Martin Eichner

**Affiliations:** 1 BODE3 Programme, University of Otago, Wellington, New Zealand; 2 HEIRU, University of Otago, Wellington, New Zealand; 3 Population Interventions Unit, Centre for Epidemiology and Biostatistics, Melbourne School of Population and Global Health, University of Melbourne, Melbourne, Australia; 4 Epimos GmbH, Dußlingen, Germany; 5 Institute for Clinical Epidemiology and Applied Biometry, University of Tübingen, Tübingen, Germany

**Keywords:** Diseases, Infectious diseases, Viral infection

## Abstract

We aimed to estimate the risk of COVID-19 outbreaks associated with air travel to a COVID-19-free country [New Zealand (NZ)]. A stochastic version of the SEIR model CovidSIM v1.1, designed specifically for COVID-19 was utilised. We first considered historical data for Australia before it eliminated COVID-19 (equivalent to an outbreak generating 74 new cases/day) and one flight per day to NZ with no interventions in place. This gave a median time to an outbreak of 0.2 years (95% range of simulation results: 3 days to 1.1 years) or a mean of 110 flights per outbreak. However, the combined use of a pre-flight PCR test of saliva, three subsequent PCR tests (on days 1, 3 and 12 in NZ), and various other interventions (mask use and contact tracing) reduced this risk to one outbreak after a median of 1.5 years (20 days to 8.1 years). A pre-flight test plus 14 days quarantine was an even more effective strategy (4.9 years; 2,594 flights). For a much lower prevalence (representing only two new community cases per week in the whole of Australia), the annual risk of an outbreak with no interventions was 1.2% and had a median time to an outbreak of 56 years. In contrast the risks associated with travellers from Japan and the United States was very much higher and would need quarantine or other restrictions. Collectively, these results suggest that multi-layered interventions can markedly reduce the risk of importing the pandemic virus via air travel into a COVID-19-free nation. For some low-risk source countries, there is the potential to replace 14-day quarantine with alternative interventions. However, all approaches require public and policy deliberation about acceptable risks, and continuous careful management and evaluation.

## Introduction

The COVID-19 pandemic spread rapidly around the world from early 2020. In response, many countries implemented control measures related to international travel with these including border closures, partial travel restrictions, exit or entry screening, and quarantine of travellers^1^. A systematic review of the effectiveness of these interventions^1^ reported that: “broadly, travel restrictions may limit the spread of disease across national borders. Entry and exit symptom screening measures on their own are not likely to be effective in detecting a meaningful proportion of cases to prevent seeding new cases within the protected region; combined with subsequent quarantine, observation and PCR testing, the effectiveness is likely to improve”.

Indeed, such measures when combined with various public health and social measures (PHSMs) such as mask use and physical distancing (sometimes including “lockdowns”), have effectively eliminated community transmission of the SARS-CoV-2 pandemic virus in a number of jurisdictions ^2^. These include those with large land borders such as China and Vietnam, but also islands such as Taiwan, Australia and New Zealand^2^. A number of small Pacific island nations that have used tight border controls have even completely avoided any community transmission of SARS-CoV-2 (e.g., Samoa, Tonga, Cook Islands).

Despite the apparent economic benefits with elimination strategies in terms of GDP impacts during 2020, when compared to countries using suppression strategies^2^, there is growing interest in how they can safely open up international travel between them to regain the social and economic benefits of quarantine-free travel (e.g., for family reunions, for business travel and for tourism). Indeed, there are arguments for how progressive expansion of “green zones” might open up the possibility of regional elimination of COVID-19 and then ultimately provide a chance of global eradication^3^.

Two countries that have had partial quarantine-free travel are Australia and New Zealand, and as of March 2021 they were in the process of expanding on this to make such travel two-way and to involve all Australian States and Territories^4^. Given such developments, we aimed to model the risk of COVID-19 outbreaks associated with international air travel from Australia to New Zealand, along with the likely impact of various control measures that could be used to minimise the risk of such outbreaks. We also aimed to consider the risk for such international travel from Japan and the United States (US) to New Zealand in scenario analyses.

## Methods

### Model design and parameters for SARS-CoV-2 and COVID-19

We used a stochastic SEIR type model with key compartments for: susceptible [S], exposed [E], infected [I], and recovered/removed [R]. The model is a stochastic version of CovidSIM which was developed specifically for COVID-19 (http://covidsim.eu; version 1.1) and built in Pascal. The stochastic simulation of the compartmental model followed the procedure described by Gillespie ^5^. If not stated otherwise, one billion simulations were run per combination of intervention strategies. Each simulation started with the random sampling of the initial infection state of 300 passengers who would then (if infected) progress in their natural history and infect others (see Supplementary Information for further detail). In a separate set of simulations, probabilistic parameter sampling within parameter uncertainty ranges (probabilistic sensitivity analysis) was also conducted (see Supplementary Information).

Work has been produced from previous versions of this model^6–8^, and the equations and their stochastic treatment are detailed in two of these outputs^9,10^. Code for the model is available online is available online at GitHub (https://github.com/nick-wilson-github/Air-travel-Covid-risk-modelling/tree/main).

The parameters were based on available publications and best estimates used in the published modelling work on COVID-19 (as known to us in February 2021).

### Prevalence of infection in Australia reflecting an outbreak

**Figure 1:**
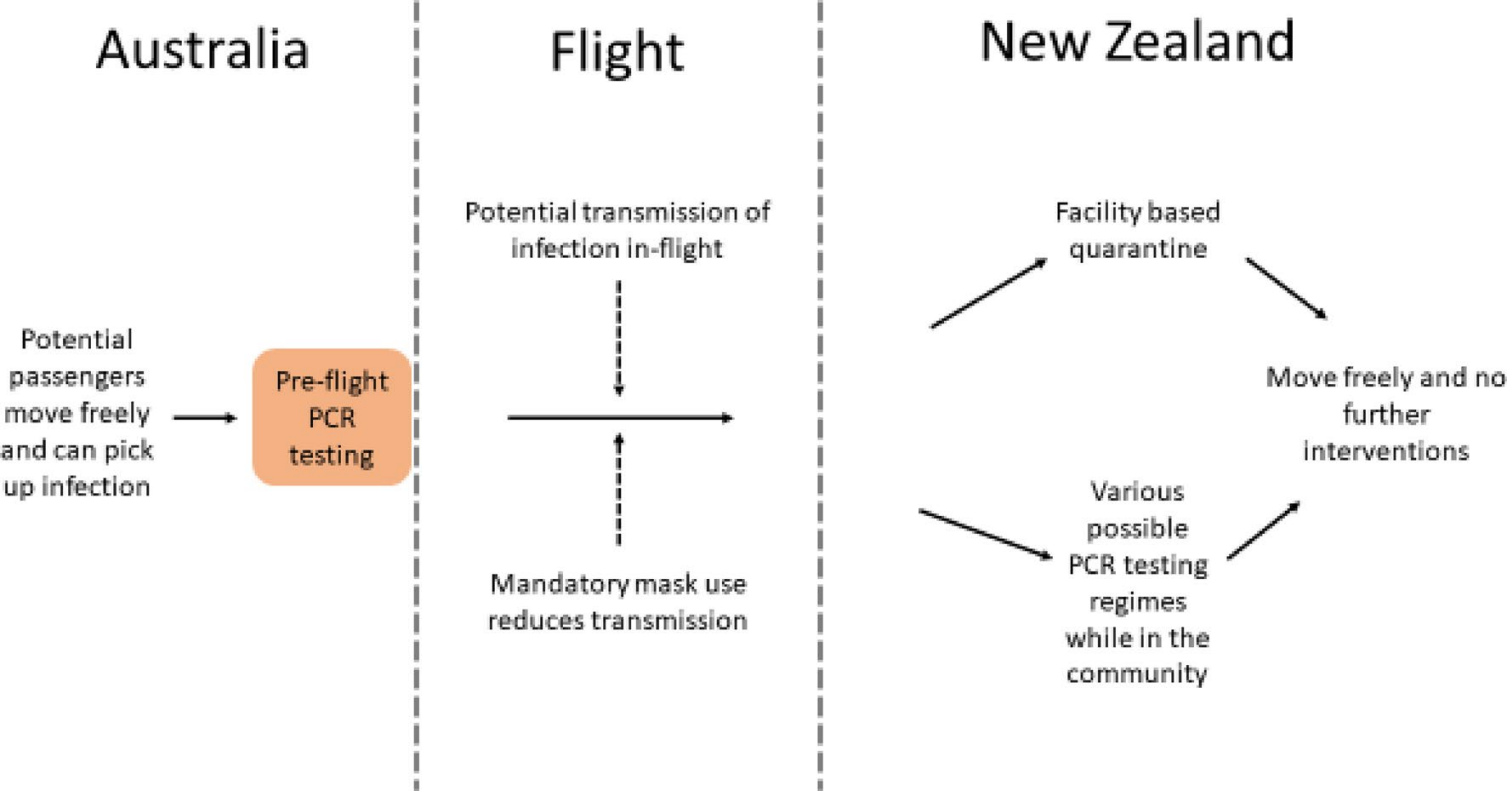
Flow diagram of the modelled movements of air travellers from Australia (when experiencing an outbreak) including the key interventions (simplified and not showing details around travellers seeking medical attention when symptomatic in New Zealand, isolation of identified cases and contact tracing).

To estimate the prevalence of SARS-CoV-2 infection in Australia as a source country we used historical data on reported cases of infection over the 1 April 2020 to 25 February 2021 period (Table 1). This does not reflect the COVID-19-free status that Australia has achieved subsequently in early 2021, but rather the situation equivalent to a substantial outbreak (actually one involving an average of 74 new cases per day). But in scenario analyses we also considered smaller outbreak sizes. For the simulations, travellers to New Zealand were randomly sampled from the Australian population. In most of our scenarios, travellers underwent pre-flight testing before boarding (see Fig. 1 and details below).

**Table 1:** Input parameters relating to the countries of origin for the travellers arriving in New Zealand (NZ).

Parameter	Australia (historical base case)*	Scenario analysis: Japan	Scenario analysis: US
Reported average new cases per day in the period 1 April 2020 to 25 February 2021 (WHO data^14,15^)	74	1289	104,594 (adjusted for under-reporting**)
Average new cases per day per million population (for the time period in the above row and using OECD population data^16^)	3.0	10.2	319.7
Estimated point prevalence per million population of SARS-CoV-2 infection on average day (assuming a 16-day long period that comprises the latent and prodromal periods plus the rest of the infectious period, see Table 2). This is what is used in the model to determine traveller risk at the time of pre-flight testing prior to departure to NZ	48	163	5115

*In the situation in Australia in early 2021 where COVID-19 elimination status had been achieved (albeit introduced cases being in quarantine and isolation in border facilities), these values are those that could be generated by a large outbreak from a border control failure. See Table 5 for scenario analyses using much lower prevalence values that would better equate to small outbreaks following border control failures in Australia. Furthermore, these historical values also partly represent cases detected at the Australian border and then managed to eliminate any risk of transmission in the community.

** Adjusted for the 1.24 times greater excess deaths in the USA relative to deaths attributable to COVID-19 as per the period 8 March 2020 and 9 January 2021^17^.

### Selection of control measures

We identified plausible control measures from the published literature^1^ and also an online review of strategies identified by an IATA Medical Advisory Group^11^. These controls are shown in Fig. 1 and Table 3.

### Air travel to New Zealand

We simulated one three-hour flight per day from Australia to New Zealand, carrying 300 passengers. A wide range of aircraft were used on this route in the pre-COVID-19 pandemic era, with common ones being the Boeing 777-200 which takes 312 passengers and the Airbus A300-300 with 297 passengers. One flight per day is a small proportion of the level of travel in the pre-pandemic time (i.e., 7.1% of the of 1,542,467 traveller arrivals from Australia to New Zealand prior to the pandemic in the year to January 2020^12^). In additional scenario analyses we also considered flights from Japan and the US (with their different population burdens of infection and longer flight times), albeit with the same assumptions for passengers as per flights from Australia to New Zealand.

### In-flight transmission risk

We identified a published review on the transmission of SARS-CoV-2 on aircraft^13^. Using this and subsequently published literature (see Supplementary Information for details), we estimated the number of hours of exposure to infected cases (number of infected people on the flights x flight hours). From this we identified two in-flight infections arising from 933 exposure-hours, giving an estimated risk of transmission per hour of flying in a plane containing an infectious person of 0.00214 (Table 2).

**Table 2:** Input parameters used for modelling the potential spread of COVID-19 infections with the stochastic version of CovidSIM (v1.1) with New Zealand as a case study.

Parameter	Value/s used	Further details for parameter inputs into the modelling
Latency period	5 days	We used the best estimate from CDC of a mean of 6 days to symptoms (i.e., the latency period plus the prodromal period)^18^. We used a standard deviation (SD) of 25% (1 day) (calculated using 16 stages; Erlang distribution)
Prodromal period	1 day	There is still uncertainty about the length of the prodromal period for COVID-19, so we used an assumed value for influenza (SD = 25%; 0.25 days, Erlang distribution)
Symptomatic period	10 days (split into 2 periods of 5 days each)	The WHO-China Joint Mission report stated that “the median time from onset to clinical recovery for mild cases is approximately 2 weeks and is 3–6 weeks for patients with severe or critical disease”^19^. But given that mild cases may have been missed in this particular assessment, we used a slightly shorter total time period of 10 days (SD = 25%; 2.5 days, Erlang distribution)
Infections that lead to sickness (symptomatic illness)	60%	We used the best estimate from CDC of 60% symptomatic and 40% asymptomatic^18^*
**Contagiousness**		
Risk of in-flight transmission	0.00214 per hour of flying	This risk was estimated for transmission from an infectious case on a flight in which there was mandated masking (i.e., masks are mandated for all international flights arriving in NZ at the time of writing in March 2020). It is the risk that an index case infects one of the fellow passengers, not the individual risk of each fellow passengers to acquire infection. See the Supplementary Information for our estimates derived from our review of the literature*
Flight duration	3 h	For the Australia to NZ flights (e.g., Sydney to Auckland). Times for flights from Japan and the US in scenario analyses are shown in Table 5*
Effective reproduction number (R_e_) in the NZ post-pandemic setting	2.5	We used the best estimate from CDC of R_0_ = 2.5^18^. We assumed for NZ that the social behaviour with elimination status was fairly similar to the pre-COVID-19 situation (i.e. negligible additional physical distancing, normal occurrence of indoor events in public settings and no routine mask use by the great majority of the population). We also assumed a population with no specific immunity to SARS-CoV-2 (acquired or via vaccination)
Relative contagious-ness in the prodromal period	100%	We assumed this was high given that the CDC estimate that 50% of transmission occurs prior to symptom onset^18^
Contagiousness after the prodromal period	100% and 50%	In the first five days of symptoms, cases were considered to be fully contagious. In the second five-day period, this was assumed to be at 50%. The latter figure is still uncertain, but is broadly consistent with one study on changing viral load^20^

*See Supplementary Information for consideration of uncertainty and probabilistic sensitivity analysis.

### Arrival in New Zealand

Upon arrival in New Zealand, travellers were either placed in supervised quarantine for various periods of days (current practice in New Zealand is for 14 days) and then released to move freely, or, as an alternative to quarantine, we considered various combinations of PCR testing (on days 1, 3 and 12). Until their last PCR test, we assumed that people could move freely around New Zealand, but were required to attend official facilities for testing and to wear a mask while in the presence of other people (Table 2). We further assumed that half of the passengers who develop symptoms during this period would report these symptoms within one day. Also, we assumed that if passengers are tested positive, or if they reported symptoms themselves, contact tracing would identify 75% of their infected contacts in New Zealand who would be isolated after another delay of one day.

### Ongoing infection transmission in New Zealand

Secondary cases were assumed to arise from spread from incoming infected travellers in the community in New Zealand. Tertiary cases were those who were infected by secondary cases before the latter were isolated, and were assumed to have the full length of their infectious period ahead of them. Some of them then can trigger an outbreak.

### Control measures assumptions

The full details on the control measures we considered are detailed in Table 3.

**Table 3:** Control measures used and their estimated efficacy in preventing SARS-CoV-2 transmission.

Control measure	Value/s used	Comment
*Pre-flight testing* using saliva (PCR) of travellers in the 24 h before departure	62.3% sensitivity	For test sensitivity we used a meta-analysis that gave a sensitivity for saliva testing (PCR) at 62.3% (95%CI: 54.5%–69.6%). This was less than for nasopharyngeal aspirate/swab and throat swab (73.3%, 95%CI: 68.1%–78.0%); and for sputum (97.2%, 95%CI: 90.3%–99.7%)^21^. We note however, that the sensitivity we used here for saliva testing may be an underestimate given other work showing equivalence between nasopharyngeal and saliva based PCR tests^22^*
*Facility-based quarantine* in NZ for travellers (current practice as per March 2021 in NZ)	7, 14 and 21 days	We ran the simulations for three different lengths of quarantine, including 14 days as used in NZ, and longer as in some other settings (e.g., 21 days in China). We assumed a high quality quarantine process where there was no cross-infection within the quarantine facility to facility workers or to other travellers in quarantine. But we assumed no additional PCR testing within quarantine. In reality, NZ combines the 14-day quarantine process with PCR testing (nasopharyngeal swab) upon arrival (“day 1”) (travellers from the majority of countries), and days 3 and 12 (all travellers). This process further helps reduce the risk by allowing for infectious individuals to be put into isolation and so reduce the risk of infecting others who are also in quarantine. But these additional benefits from testing are probably outweighed by the limitations in NZ’s processes that have resulted in various failures of the quarantine/isolation facility system that utilises converted hotels^23^
*Testing instead of facility-based quarantine*: PCR test for SARS-CoV-2 at various times	The time course of sensitivity values from Kucirka et al. was used	We used the results of a study^24^ which fitted a Bayesian hierarchical logistic regression model for test sensitivity. This meant for example, at day 4 after infection, 67% of test results were false negatives. This decreased to 20% on day 8 and then increased after this e.g., up to 66% on day 21. For cases who already recovered before their PCR test, we use the final value reported by Kucirka et al. (i.e., 34% sensitivity). In the days after arrival and before the next PCR test, we assume that people act normally and so can potentially spread infection to the NZ public (albeit with mask use when with other people as per the details below). For more details, see Supplementary Information In the absence of relevant data, we had to assume test result independence i.e., a false negative for a test was not correlated with a false negative for a later test. If both results were negative, we assumed no further follow-up. We considered a wide range of different timing options for PCR tests after arrival in NZ (see the “Results”)
*Contact tracing* if (i) a scheduled PCR test is positive or (ii) if people develop symptoms and seek medical attention (see below)	80% of infected contacts are traced and isolated within 48 h	We used performance data for the cluster of cases in Auckland in August 2020 where the official estimate was 80% of contacts contacted within 48 h (as reported by the Prime Minister)^25^. We divided this into 60% within the first 24 h and 20% in the next 24 h. Of note is that variable performance for contact tracing has been reported for NZ at other times in August 2020, with 86% of contacts traced in 48 h at one point^26^*
The proportion of infected travellers who when they develop any symptoms *seek medical attention* (i.e., they are in the 60% who will ever develop symptoms)	50% (self-reporting occurs on average 1 day after symptom onset)	We assumed that this proportion is somewhat higher than that for the general community (see below) on the assumption that these travellers would be provided information on the flight and on arrival in NZ on the critical importance of seeking medical attention if they develop any symptoms. They would also be told that such medical attention would be provided free of charge. We assumed PCR confirmation of self-reported symptoms and if a positive test, then we assumed case isolation and potentially triggering contact tracing Of note is that routinely in NZ, 39.5% of people with “fever and cough” symptoms seek medical attention, as reported by the NZ Flutracking surveillance system^27^. This is very similar to international estimates for people with influenza who seeking medical attention at 40% e.g., as used in other modelling^6^
*Quarantine* of traced contacts	1 day after detection of index cases	Traced contacts are assumed to be effectively quarantined with no further spread of infection
Mandatory *mask use* by incoming travellers up to the time of their final PCR test in the NZ community	66% transmission reduction	We used the results of the most recent meta-analysis we could identify which involved 29 studies on infection with SARS-CoV-2, SARS, or MERS^28^. It reported that “type N-95 masks (corresponding approximately to FFP-2), surgical masks, or similar multilayer cotton masks can greatly reduce the infection risk for the wearers (RR 0.34 [0.26; 0.45]).”*

*See Supplementary Information for consideration of uncertainty and probabilistic sensitivity analysis.

## Results

Our baseline results used historical data for Australia (equivalent to a border failure resulting in an outbreak generating 74 new cases/day in an otherwise COVID-19-free Australia) and one flight per day to New Zealand with no interventions in place (other than mandated masks on flights). This resulted in the median time to an outbreak in New Zealand being 0.2 years (95% range of simulation results: 3 days to 1.1 years) or after a mean of 110 flights (Table 4). However, the risk progressively declined with the addition of pre-flight testing, testing when in New Zealand, mask use up to the last test, with symptom reporting, and contact tracing. Given all these (with testing on days 1, 3 and 12 in New Zealand), the median time to an outbreak was extended to 1.5 years (20 days to 8.1 years; or a 37% chance per year; or after a mean of 802 flights) (Table 4). Mask wearing by travellers when in the New Zealand community (up to the time of their last test), had a much larger impact than various aspects of the proportion self-reporting symptoms and contact tracing performance. However, 14 days quarantine was a more effective strategy with a median time to an outbreak of 4.9 years (66 days to 26.2 years; mean of 2,594 flights); albeit combined with a pre-flight test. Even better was 21 days of quarantine which was associated with virtually a zero risk of an outbreak ever occurring.

**Table 4:** Results of the simulations of the baseline risk (no interventions) and for multi-layered packages of interventions to prevent COVID-19 outbreaks in New Zealand (NZ) (assuming a historical level of infection in Australia that was equivalent to a border failure resulting in an outbreak generating 74 new cases/day (as per Table 1) and mandatory mask use on international flights).

Strategy	Pre-flight saliva test sensitivity	Quarantine of travellers	PCR tests for travellers (day 1 is arrival day)*	Traced contacts after positive PCR test	Prevented infections in NZ while travellers wear masks**	Symptomatic travellers who self-report symptoms ***	Traced contacts after self-reporting of symptoms	Annual risk of outbreak in NZ	Median waiting time until next outbreak occurs (95% range)	Mean number of flights to create one outbreak
No PCR tests, no quarantine	–	–	–	–	–	–	–	96.4%	0.2 years (y) (3 days [d]–1.1 y)	110
	62.3%	–	–	–	–	–	–	88.4%	0.3 y (4 d–1.7 y)	170
**Addition of PCR tests after arrival in NZ (in addition to pre-flight testing)**										
PCR tests	62.3%	–	day 1	–	–	–	–	80.3%	0.4 y (6 d–2.3 y)	225
	62.3%	–	days 1 + 3	–	–	–	–	65.4%	0.7 y (9 d–3.5 y)	344
	62.3%	–	days 1 + 3	75%	–	–	–	65.3%	0.7 y (9 d–3.5 y)	345
	62.3%	–	days 1 + 3	75%	–	50%	75%	58.9%	0.8 y (10 d–4.2 y)	411
	62.3%	–	days 1 + 3	75%	66%	–	–	49.8%	1 y (13 d–5.4 y)	530
	62.3%	–	days 1 + 3	75%	66%	50%	75%	42.8%	1.2 y (17 d–6.6 y)	653
	62.3%	–	days 1 + 3 + 12	–	–	–	–	63.9%	0.7 y (9 d–3.6 y)	359
	62.3%	–	days 1 + 3 + 12	75%	–	–	–	63.5%	0.7 y (9 d–3.7 y)	362
	62.3%	–	days 1 + 3 + 12	75%		50%	75%	57.2%	0.8 y (11 d–4.4 y)	431
	62.3%	–	days 1 + 3 + 12	75%	66%	–	–	43.0%	1.2 y (16 d–6.6 y)	650
	62.3%	–	days 1 + 3 + 12	75%	66%	50%	75%	36.6%	1.5 y (20 d–8.1 y)	802
**Facility-based quarantine (in addition to pre-flight testing)**										
Quarantine	62.3%	7 days	–	–	–	–	–	67.8%	0.6 y (8 d–3.3 y)	322
	62.3%	14 days	–	–	–	–	–	13.1%	4.9 y (66 d–26.2 y)	2594
	62.3%	21 days	–	–	–	–	–	0.0%	n.a.	745,000

One billion stochastic simulations were run for each intervention strategy. Result values typically rounded to three meaningful digits. Travellers are allowed to move freely in NZ from arrival to the last PCR test or after being released from quarantine (see Fig. 1).

*A range of days were considered, but the 1 + 3 + 12 day option is the one typically used for travellers to NZ (albeit combined with quarantine for all travellers to NZ).

**Prevention of secondary infections due to wearing of masks by travellers when in NZ up to the time of the last PCR test.

***The given fraction of passengers who report having developed symptoms while staying in NZ to the health system; they are assumed to be isolated one day after symptom onset and contact tracing may occur after this; traced contacts are PCR tested and isolated after another delay of one day.

Table 5 details a range of scenario analyses including if Australia’s infection prevalence was 10 times lower than in the historical base case (i.e., approximating if Australia had a small outbreak from a border failure with around seven new cases per day). For this particular scenario and with no interventions the annual risk of an outbreak was 28.3% and had a median time to an outbreak of 2.1 years. For an even lower prevalence (representing only two new community cases per week in the whole of Australia), the annual risk of an outbreak with no interventions was 1.2% and had a median time to an outbreak of 56 years. But these risks would increase with higher travel volumes as per the scenario of a 20-fold increase in travel from Australia.

**Table 5:** Scenario analyses covering different source countries, SARS-CoV-2 infection burdens and flight volumes (with the base case for comparison).

Scenario/country setting	Intervention	Annual risk of outbreak in NZ	Median waiting time until next outbreak occurs (95% range)****	Mean number of flights to create one outbreak^#^
*Australia to NZ (base case)* with the historical prevalence in Australia of 48/million (Table 1), 1 flight per day 3 h flight time	None	96.4%	76 d (3 d–407 d)	110
	2 PCR in NZ*	42.8%	1.2 y (17 d–6.6 y)	652
	3 PCR in NZ**	36.6%	1.5 y (20 d–8.1 y)	800
	Quarantine***	13.1%	4.9 y (66 d–26.2 y)	2593
Australia to NZ, as per the historical base case above but with *20 flights per day* (slightly more than the pre-pandemic level)	None	100%	4 d (1 d–22 d)	110
	2 PCR in NZ*	100%	23 d (1 d–122 d)	652
	3 PCR in NZ**	100%	28 d (1 d–150 d)	800
	Quarantine***	100%	90 d (3 d–480 d)	2593
Australia to NZ, as per base case but with *1/10 of the historical base case prevalence* in Australia: 4.8 / million; (equivalent to a small outbreak from a border failure averaging 7.4 new cases per day)	None	28.3%	2.1 y (28 d–11.1 y)	1099
	2 PCR in NZ*	5.4%	12.4 y (165 d–65.9 y)	6520
	3 PCR in NZ**	4.5%	15.2 y (203 d–80.9 y)	8005
	Quarantine***	1.4%	49.2 y (1.8–262 y)	25,928
Australia to NZ, as per the base case but with the prevalence in Australia closer to *March 2021 settings* and a border failure generating 2 new cases per week; prevalence 0.18 / million^##^	None	1.2%	56 y (2–297 y)	29,420
	2 PCR in NZ*	0.2%	331 y (12–1760 y)	174,114
	3 PCR in NZ**	0.2%	406 y (15–2161 y)	213,809
	Quarantine***	0.1%	1,314 y (48–6991 y)	691,710
*Japan to NZ***,** with a prevalence of 163 / million in Japan (Table 1). 1 flight per day, 10.6 h flight time	None	100%	23 d (1–124 d)	34
	2 PCR in NZ*	83.0%	143 d (5 d–2.1 y)	206
	3 PCR in NZ**	77.0%	173 d (6 d–2.5 y)	249
	Quarantine***	34.3%	1.7 y (22 d–8.8 y)	870
*US to NZ,* with a prevalence of 5,115 / million in the US (Table 1). 1 flight per day, 13 h flight time	None	100%	1 d (1–6 d)	2
	2 PCR in NZ*	100%	5 d (1–27 d)	7
	3 PCR in NZ**	100%	6 d (1–32 d)	9
	Quarantine***	100%	20 d (1–109 d)	29

For each intervention strategy, 1 billion stochastic simulations were run for “Australia base case”, “Australia 20 flights/day” and “Australia 1/10 prevalence”; 100 million simulations for all other country settings.

*Pre-flight saliva test; 2 PCR tests in NZ (on days 1 and 3); until second PCR, passengers wear masks in NZ and self-report symptoms (contacts are traced and quarantined).

**Pre-flight saliva test; 3 PCR tests in NZ (on days 1, 3 and 12); until third PCR, passengers wear masks in NZ and self-report symptoms (contacts are traced and quarantined).

***Pre-flight saliva test; quarantine of all passengers in NZ for 7 days.

^#^The median waiting time until an outbreak occurs only refers to the time until a plane lands which will cause an outbreak in NZ; further days are needed until a passenger infects somebody, until the infection spreads in NZ to many others, and until an outbreak is officially declared. Passengers who are found positive during one of the NZ based PCR tests or traced and detected secondary cases do not trigger the declaration of an outbreak. Here, an outbreak is assumed to be the out-of-control spread of SARS-CoV-2 which will reach many cases if not prevented by major interventions.

^##^Due to the very low prevalence, no additional simulations were run for this scenario, but the outbreak probabilities per flight were linearly extrapolated from the corresponding base case simulations (based on 1 billion flights each); the reported output was calculated from the obtained probability per flight as detailed in the Supplementary Information.

The high prevalence of infection for the US meant that, even with quarantine, the median time to an outbreak was only 20 days, or after only a mean of 29 flights (Table 5). But the equivalent values for Japan were much longer (1.7 years) and larger (870 flights).

The stochastic simulations using base case parameters presented in this main text were also directly compared with a probabilistic sensitivity analysis (PSA) involving random sampling from the parameter distributions (results in the Supplementary Material: Table S3). This comparison showed almost identical results from the two approaches.

## Discussion

### Main findings and interpretation

This analysis examined the risk of COVID-19 outbreaks in a COVID-19-free nation (New Zealand), if there was air travel from a low-prevalence country (e.g., if Australia experienced various sizes of outbreaks from border failure and lost its COVID-19 “elimination status”). Using the historical data for Australia (equivalent to an outbreak with 74 new cases per day) and no interventions, we estimated that there would be such an outbreak of COVID-19 in New Zealand after a median time of only two to three months. Fortunately, the multi-layered packages of interventions we modelled reduced this risk to much lower levels. Indeed, without quarantine, the use of a package of measures (pre-flight testing, PCR testing in New Zealand, mask use and contact tracing) could reduce the risk to potentially tolerable levels—if health authorities had confidence in the application of these measures in the real world (e.g., adherence with mask use by travellers and minimal defaulting on testing when in the New Zealand community).

More realistically however, for the situation in early 2021 where Australia has effectively eliminated COVID-19 in the community, is to consider the smaller outbreak scenarios. These would suggest a low risk of an outbreak in New Zealand (e.g., only a 1.2% annual risk with no interventions and a small outbreak of two new cases per week in Australia). But even at this low risk level, New Zealand health authorities might still wish to promote existing digital tools to incoming travellers so as to facilitate rapid outbreak control (e.g., New Zealand encourages QR code scanning when entering buildings and buses etc.).

But for travellers from Japan, the risks are much higher than for travellers from Australia and quarantine would probably remain appropriate. Whereas for travellers from the US the very high risk might suggest that tighter travel restrictions are more appropriate until epidemic spread was reduced. Alternatively processes such as pre-flight quarantine could be considered for US travellers.

Our findings have some compatibility with those of another modelling study which reported that various interventions (including pre-flight and post-flight testing and five day quarantine) would reduce spread of SARS-CoV-2 associated with domestic travel within the US^29^. But the package of interventions modelled were less intensive than in our model and at best reduced the number of “infectious days” in the modelled cohort by only 70%.

It is likely that all these travel-related risks will decline as vaccinations are provided to: (i) the population of source countries; (ii) travellers (in the weeks prior to travel); and (iii) the population of recipient countries. As such, future modelling should consider vaccination coverage and vaccination effectiveness in preventing transmission. Future modelling should also ideally factor in costs so that cost-effectiveness ratios can be calculated for various intervention packages and their marginal adjustments. The relevance of these costs to policy-makers might also be impacted by who is paying. For example, if all incoming travellers were charged a COVID-19 levy and aspects of the system could be made user-pays (indeed some charges are already used in the New Zealand border system for quarantine).

Ultimately, there is also a need for full cost–benefit analyses which consider the benefits of increased travel to the recipient society and economy—along with the risks of outbreaks that need to be rapidly controlled or else pose a risk of lockdown measures being required.

### Study strengths and limitations

This is the first such study (that we know of), to model such interventions in the context of preventing the re-introduction of SARS-CoV-2 into a country that has eliminated it. We were also able to consider a wide range of control interventions and to package these in multiple layers of defence. Nevertheless, there is quite high uncertainty around some of the parameters we used. For example, the prevalence of infection within source countries is, in reality, highly heterogeneous (by age group, social group and locality) and will vary over time. Indeed, Australia in early 2021 had effectively eliminated SARS-CoV-2 transmission with just occasional small and rapidly controlled outbreaks arising from border control failures^23^. Furthermore, our data on the effectiveness of masks on aircraft was based on a limited amount of real world experience (i.e., only eight flights with cases on board and mask mandates in place). We also did not model infection amongst air crew due to the complexity of their international movements and different control measures used with this occupational group, but note that these personnel have been a rare cause of COVID-19 related border failures in New Zealand to date.

Another limitation is that we did not account for a small proportion of travellers who might cancel their flight to New Zealand if becoming symptomatic after infection with SARS-CoV-2. Furthermore, the probability of symptomatic illness amongst travellers will probably be different than in the general population (e.g., if travel is dominated by younger adults who are less concerned with pandemic risks). We also assumed full adherence to testing regimens within the New Zealand community, though potentially this could be achieved if large fines were imposed or if travellers paid a large financial bond that was only redeemed after full adherence.

Finally, we assumed quarantine only failed due to a tiny proportion of cases having very long incubation periods. In reality, however, a country like New Zealand has had failures with its COVID-19 quarantine system with facility workers and other travellers becoming infected^23^. This is because it uses re-purposed hotels instead of purpose-built facilities and does not confine the travellers to their rooms (i.e., there are shared corridors, lifts, exercise areas and smoking areas). Given all such issues and ongoing improvements in knowledge of the transmission dynamics of SARS-CoV-2, this type of modelling work should be regularly revised and be performed using different types of models.

## Conclusions

This modelling study suggests that the risk of an outbreak in a previously COVID-19-free country is extremely dependent on the source country of the incoming travellers. In the situation of Australia experiencing a large outbreak, the risk could potentially be reduced to tolerable levels with a package of multi-layered interventions (particularly with repeated testing and mask use) and no quarantine. Nevertheless, quarantine is likely to remain important where the source country has high disease burdens. However, all approaches require public and policy deliberation about acceptable risks, and continuous careful management and evaluation.

## Supplementary Information

Supplementary Information.
