# Supplementary material for: Estimating the impact of control measures to prevent outbreaks of COVID-19 associated with air travel into a COVID-19-free country
Source: Sci Rep. 2021 May 24;11:10766. doi: 10.1038/s41598-021-89807-y (PMC8144219; doi:10.1038/s41598-021-89807-y)
Supplement: Supplementary file 1 — Supplementary Information. [file 41598_2021_89807_MOESM1_ESM.docx]

**Supplementary Material**

**“Estimating the Impact of Control Measures to Prevent Outbreaks of COVID-19 Associated with Air Travel into a COVID-19-free country”**

Nick Wilson^1 2 *^, Michael G Baker^2^, Tony Blakely^3^, Martin Eichner ^4 5^

^1^ BODE^3^ Programme, University of Otago Wellington, New Zealand

^2^ HEIRU, University of Otago Wellington, New Zealand

^3^ Population Interventions Unit, Centre for Epidemiology and Biostatistics, Melbourne School of Population and Global Health, University of Melbourne, Australia

^4^ Epimos GmbH, Germany

^5^ Institute for Clinical Epidemiology and Applied Biometry, University of Tübingen, Germany

* Corresponding author: Prof Nick Wilson, Email: nick.wilson@otago.ac.nz

**Estimation of risk of SARS-CoV-2 transmission on aircraft flights where masks are mandated**

We extracted data from the only published review we identified^1^. We supplemented these data with findings from a literature search of PubMed conducted on 28 February 2021 (search terms: aircraft AND transmission AND (COVID-19 OR SARS-CoV-2)). Results are shown in Table S1. In the analysis we excluded data from evacuation flights where top quality (N95 or equivalent) masks were used (but these are still shown in Table S1 to provide further context). Unfortunately many of the identified studies did not provide detail on mandated mask use (see Table S1 footnotes).

The estimated overall risk was calculated as that for an infected person infecting one other per flight. This approach was considered more meaningful than adjusting the risk per people on the flight because of the evidence for infection risk being higher for those in close seating proximity to the index case^1^ ^2^.

**Table S1: Our analysis of extracted data from a published review^1^ on in-flight transmission of SARS-CoV-2 and additional searches of published literature (all for only flights where mask use was mandated and excluding those involving N95 masks*)**

| **Flight** | **Number of infected cases on the flight** | **Cases arising from in-flight transmission** | **Comments** |
| --- | --- | --- | --- |
| ***Data from a review^1^*** | |  |  |
| Flight D | 25 | 2 | 10.7 hour flight |
| Flight G | 2 | 0 | Evacuation flight but passengers using surgical masks. 19 hour flight |
| Flights N to R (5 flights) | 58 | 0 | 5 flights with a total of 1500 to 2000 passengers (each 10.7 hours) |
| ***Data from other identified work*** | |  |  |
| Ng et al. 2020^3^ | 2 | 0 | 4.8 hour flight |
| **Total (all of the above)** | **87** | **2** | To calculate the risk to passengers on mandated mask flights with infected people we estimated the number of hours of exposure (number of index cases on the flight x flight hours). This indicated 2 infections arising from 933 exposure-hours, giving a risk of transmission per hour of flying of 0.002. For the uncertainty around this value, we applied a lognormal distribution (SD = 0.0019). These were the values used in the modelling. |
| ***Background – data from studies of evacuation flights with mandated N95 masks (provided for additional context but not used in deriving parameters for our modelling)*** | | | |
| Flight E^1^ | 6 | 1 | Transmission classified as "likely" |
| Bae et al. 2020^4^ | 3 | 1 | Considered “likely” transmission. This was for a separate flight but detailed by the same authors as per Flight E in the row above. |
| Cornelius et al. 2020^5^ | Many (see comments) | 0 (crew infected) | “Over 2,000 distinct patients all of whom were either COVID-19 positive, persons under investigation (PUIs), or individuals who were asymptomatic were flown on 39 missions without infection of any transporting HHS air medical evacuation crews.” |

* We excluded those flights with mask use where the results were more equivocal (ie, Flights J and L in the review, where in-flight transmission was described as “possible”). Also in one of these flights (J) the person possibly infected on the flight had removed their mask to talk for 1 hour. Other studies excluded from this analysis were those with incomplete information on mask requirements or mask use^6^ ^7^ ^8^ ^9^ ^10^ ^11^) or where mask use was reported to be incomplete^12^.

**Sensitivity of PCR tests during the course of infection**

Kucirka *et al.*^13^ provide in their Figure 2 estimates on the time-course of the fraction of false-negative PCR results. We have reproduced their curve and translated it into a time-course that gives the test sensitivity for infected individuals, depending on the time since they were exposed to infection. As our simulations are not individual-based, but stochastic representations of a compartmental model, we cannot exactly know the time since exposure for an infected individual who is in a given state of infection (which is represented by one of the 16 latent stages, the 16 prodromal stages, the 16 early and the 16 late infections stages). In order to match these 4x16 infection stages, we ran a specific set of one million stochastic simulations with one infected individual each, recorded the time when this individual entered or left any one of these stages and finally calculated the individual’s middle time point (since infection) for each of the 4x16 infection stages. In the next step, we recorded the sensitivity values that corresponded to the recorded 4x16 simulated time points of the individual, leading to one million simulation-matched values for each of the 4x16 infection stages. In a final step, we averaged over these one million values, obtaining 4x16 sensitivity values for the infection stages that we used in the simulations.

**Additional detail on calculations (eg, probability of an outbreak)**

Passengers are randomly sampled from their departure country, taking into consideration the local prevalence of infection. If an infected passenger is sampled, a random number is calculated to determine how far back the infection occurred and, thus, in what epidemiologic state the passenger is when entering the airport. From this point on, the progress of the passengers’ infection and the passing on of the infection to others is simulated by a compartmental stochastic model. The model is based on the differential equations given elsewhere^14 15^, but is treated stochastically as explained in Gillespie^16^. The exit screening of passengers bars a percentage of infected passengers who are at least in their prodromal period from entering the plane. Infected passengers who enter the plane progress in their infected period on board (and later in New Zealand). If they are already infectious or become infectious on board, they can infect others. After entering New Zealand, the passengers take a PCR test (in most scenarios) which can detect some of the infected passengers and prevent them from entering the community (they are assumed to be transferred to isolation facilities). Undetected infected individuals who enter New Zealand and who are already infectious or become infectious in New Zealand can infect local people. It the passengers self-report systems or if they are found positive at a later PCR test, most of the infected local people can be traced and isolated, but in some rare cases, some of them may already have generated tertiary infections. The passengers’ self-reporting of symptoms and their wearing of masks in New Zealand (if applicable) lasts until their last PCR test and is discontinued thereafter. Each simulation of a flight to New Zealand ends with a number (i = 0, 1, 2 ...) of undetected infected individuals in New Zealand. In a separate series of simulations which always starts with a single newly infected individual, the probability that this one individual triggers a major outbreak (defined as at least 100 infections in New Zealand; no interventions like masks etc. are assumed in this second series of simulations). The fraction f of such simulations which lead to a major outbreak is calculated and then applied to the outcome of the first simulations in which a flight resulted in i undetected infected people in New Zealand. The probability that the flight results in a major outbreak is then calculated as x = 1-(1-f)^i^. The expected number of major outbreaks for all 10^9^ simulated flights is obtained by adding up the probabilities x for all flights. Dividing the expected number of major outbreaks by the number of simulated flights, we obtain the probability p that one flight generates a major outbreak. This probability p can be used to derive various other output (all following calculations assume that one flight occurs per day; they have to be adjusted if the flight frequency differs): (a) The average waiting time in days until a major outbreak occurs is 1/p. (b) The median waiting time in days until a major outbreak occurs is

-ln(0.5)/p. (c) 95% of major outbreaks are expected to occur between -ln(0.025)/p and -ln(0. 975)/p days. (d) The probability that an outbreak occurs within a year is 1-(1-p)^365^. The number of flights per major outbreak is 1/p.

**Distributions of key parameters and comparisons with a probabilistic sensitivity analysis**

For each single simulation, parameters were sampled independently from the distributions shown below.

**Table S2: Distributions used for the random sampling of parameter values**

| **Parameter** | **Distribution** | **Sample of 1000 values** |
| --- | --- | --- |
| Sensitivity of  pre-flight saliva (PCR) test  [%] | Normal distribution* mean 62.3 SD 3.85 | 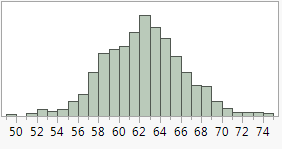 |
| Flight  duration  [hours] | Normal distribution**  mean 3 hours SD 0.3 hours (10% of mean) | 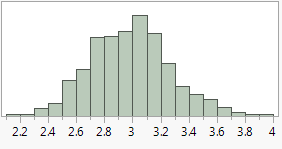 |
| In-flight risk of infection [per hour] | Lognormal distribution* mean 0.002185/hour  SD 0.0018503/hour | 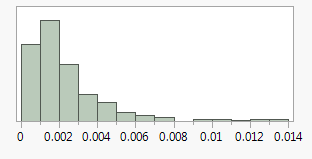 |
| Effective reproduction number in NZ | Normal distribution**  mean 2.5 SD 0.25 (10% of mean) | 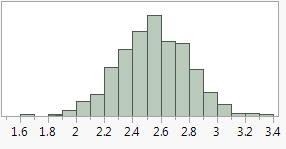 |
| Efficacy of wearing masks in NZ  [%] | Normal distribution*  mean 66% SD 5.4% | 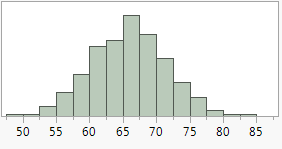 |
| Probability that infection leads to  disease  [%] | Normal distribution**  mean 60% SD 6% (10% of mean) | 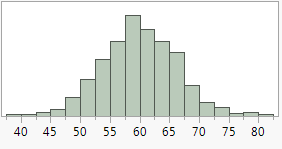 |
| Probability that a sick  passenger  self-reports  symptoms  [%] | Normal distribution**  mean 50% SD 5% (10% of mean) | 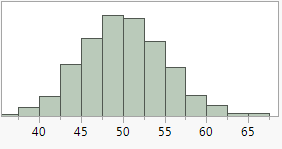 |
| Probability that an  infection  in NZ is traced [%] | Normal distribution**  mean 80% SD 8% (10% of mean) | 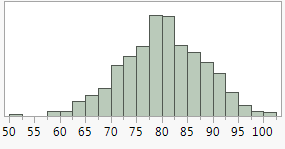 |
| Average duration of  tracing  [days] | Normal distribution**  mean 2 days SD 0.2 days (10% of mean) | 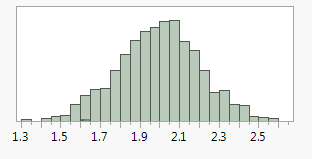 |

* Reproducing the reported 95% confidence interval (see Table 2 in the main manuscript)
** Arbitrary (authors’ judgement)

**Table S3: Results of simulations whose parameters were either fixed (“Base case”) or independently and randomly sampled from the distributions shown in Table S2 (probabilistic sensitivity analysis; “PSA”). For each setting, 1 billion simulations were run and evaluated. The distributions shown in the table visualise the frequency of the number of simulations where 1, 2, 3 ... infections in NZ were caused by incoming travellers (the frequency of 0 infections is not shown because its height would dwarf the other bars).**

| Base case | PSA |
| --- | --- |
| **No interventions** | |
| 9,514,285 simulations with at least one infection in NZ with a total of 21,801,663 infections in NZ  (2.29 infections per successful introduction) | 9,485,933 simulations with at least one infection in NZ with a total of 21,783,799 infections in NZ  (2.38 infections per successful introduction) |
| Duration until outbreak: 0.21 (0.01-1.12) years*  Outbreak probability: 96.4%/year  110 flights needed on average for one outbreak | Duration until outbreak: 0.21 (0.01-1.12) years*  Outbreak probability: 96.3%/year  111 flights needed on average for one outbreak |
|  |  |
| **2 PCR tests with full intervention** | |
| 1,649,052 simulations with at least one infection in NZ  with a total of 2,722,334 infections in NZ  (1.65 infections per successful introduction) | 1,639,128 simulations with at least one infection in NZ  with a total of 2,720,063 infections in NZ  (1.54 infections per successful introduction) |
| Duration until outbreak:1.24 (0.05-6.60) years*  Outbreak probability: 42.8%/year  653 flights needed on average for one outbreak | Duration until outbreak:1.25 (0.05-6.67) years*  Outbreak probability: 42.5%/year  660 flights needed on average for one outbreak |
|  |  |
| **3 PCR tests with full intervention** | |
| 1,360,179 simulations with at least one infection in NZ with a total of 1,953,362 infections in NZ  (1.44 infections per successful introduction) | 1,353,584 simulations with at least one infection in NZ with a total of 1,957,365 infections in NZ  (1.40 infections per successful introduction) |
| Duration until outbreak:1.52 (0.06-8.10) years*  Outbreak probability: 36.6%/year  802 flights needed on average for one outbreak | Duration until outbreak: 1.54 (0.06-8.18) years*  Outbreak probability 36.3%/year  809 flights needed on average for one outbreak |
|  |  |
| **Quarantine for 14 days** | |
| 426,134 simulations with at least one infection in NZ with a total of 530,127 infections in NZ  (1.24 infections per successful introduction) | 426,238 simulations with at least one infection in NZ with a total of 530,583 infections in NZ  (1.23 infections per successful introduction) |
| Duration until outbreak:4.93 (0.18-26.22) years*  Outbreak probability: 13.1%/year  2594 flights needed on average for one outbreak | Duration until outbreak: 4.95 (0.18-26.33) years*  Outbreak probability 13.1%/year  2606 flights needed on average for one outbreak |
|  |  |

*The median duration and the 95% simulation interval is given for the waiting time until a major outbreak occurs in NZ.

**References**

1 Freedman, D. O. & Wilder-Smith, A. In-flight transmission of SARS-CoV-2: a review of the attack rates and available data on the efficacy of face masks. *J Travel Med* **27**, doi:10.1093/jtm/taaa178 (2020).

2 Harries, A. D., Martinez, L. & Chakaya, J. M. SARS-CoV-2: how safe is it to fly and what can be done to enhance protection? *Trans R Soc Trop Med Hyg* **115**, 117-119, doi:10.1093/trstmh/traa106 (2021).

3 Ng, O. T. *et al.* SARS-CoV-2 Infection among Travelers Returning from Wuhan, China. *The N Engl J Med* **382**, 1476-1478, doi:10.1056/NEJMc2003100 (2020).

4 Bae, S. H. *et al.* Asymptomatic Transmission of SARS-CoV-2 on Evacuation Flight. *Emerg Infect Dis* **26**, 2705-2708, doi:10.3201/eid2611.203353 (2020).

5 Cornelius, B. *et al.* Mass Air Medical Repatriation of Coronavirus Disease 2019 Patients. *Air Med J* **39**, 251-256, doi:10.1016/j.amj.2020.04.005 (2020).

6 Murphy, N. *et al.* A large national outbreak of COVID-19 linked to air travel, Ireland, summer 2020. *Euro Surveill* **25**, doi:10.2807/1560-7917.ES.2020.25.42.2001624 (2020).

7 Qian, G. Q. *et al.* Epidemiologic and Clinical Characteristics of 91 Hospitalized Patients with COVID-19 in Zhejiang, China: A retrospective, multi-centre case series. *QJM* (E-publication 18 March), doi:10.1093/qjmed/hcaa089 (2020).

8 Schwartz, K. L. *et al.* Lack of COVID-19 transmission on an international flight. *CMAJ* **192**, E410, doi:10.1503/cmaj.75015 (2020).

9 Yang, N. *et al.* In-flight transmission cluster of COVID-19: a retrospective case series. *Infect Dis (Lond)* **52**, 891-901, doi:10.1080/23744235.2020.1800814 (2020).

10 Eldin, C., Lagier, J. C., Mailhe, M. & Gautret, P. Probable aircraft transmission of Covid-19 in-flight from the Central African Republic to France. *Travel Med Infect Dis* (E-publication 5 April), 101643, doi:10.1016/j.tmaid.2020.101643 (2020).

11 Pavli, A. *et al.* In-flight transmission of COVID-19 on flights to Greece: An epidemiological analysis. *Travel Med Infect Dis* **38**, 101882, doi:10.1016/j.tmaid.2020.101882 (2020).

12 Swadi, T. *et al.* Genomic Evidence of In-Flight Transmission of SARS-CoV-2 Despite Predeparture Testing. *Emerg Infect Dis* **27**, doi:10.3201/eid2703.204714 (2021).

13 Kucirka, L. M., Lauer, S. A., Laeyendecker, O., Boon, D. & Lessler, J. Variation in false-negative rate of reverse transcriptase polymerase chain reaction-based SARS-CoV-2 tests by time since exposure. *Annal Intern Med* (E-publication 19 April), doi:10.7326/M20-1495 (2020).

14 Wilson, N. *et al.* When can elimination of SARS-CoV-2 infection be assumed? Simulation modelling in a case study island nation. medRxiv 2020;(20 May). <https://medrxiv.org/cgi/content/short/2020.05.16.20104240v1>.

15 Wilson, N. *et al.* Detecting the re-emergent COVID-19 pandemic after elimination: modelling study of combined primary care and hospital surveillance. *N Z Med J* **133**, 28-39 (2020).

16 Gillespie, D. A general method for numerically simulating the stochastic time evolution of coupled chemical reactions. *J Comput Phys* **22**, 403-434 (1976).
